# Infantile Paralysis

**Published:** 1893-09-23

**Authors:** B. Rogers

**Affiliations:** Physician to the Bristol Hospital for Children and Women


### INFANTILE PARALYSIS.

By B. Rogers, B.A., M.D., Oxon., Physician to the Bristol Hospital for Children and Women.

One of the most serious diseases that childhood is liable to is that known as infantile paralysis. It is not that fatal results often follow so much as the paralysis and deformities that subsequently appear, for they are often of so severe a nature that the patient is incapable of following any active vocation, and is doomed to a lifelong deformity rendering him incapable from following many professions and callings.

What circumstances or causes are most potent in producing an attack it is difficult to say. In many cases we find no definite history of any exposure, and it is only in the minority that the disease appears to follow an attack of one of the acute specific fevers, or from too early attempts at walking, or from the effects of a cold or chill, or from troubles during dentition. From whatever cause the disease arises, the symptoms are the same, though it is more than probable that a certain number of cases in the acute stage are overlooked or wrongly diagnosed from the indefiniteness of the early symptoms, or from their resemblance to those ushering in other diseases. It is probable that rapidly fatal cases are often registered as dying from other causes, as convulsions, &c. When the acute stage has passed off and the paralysis is well marked, a definite decision on the nature of the case is by no means difficult. For convenience the progress of the disease has been spoken of as taking place in four stages. The division is arbitrary but useful. In the first stage a child, to all appearances healthy in every way, is suddenly taken with a febrile attack of more or less severity. The symptoms are not unlike those that are seen before the development of the rash in the acute specific fevers. The child is ill; there is fever, vomiting, muscular twitching, and sometimes serious disturbance, and after a short time a limb, or it may be two, or at least a few muscles are found flaccid, limp, and powerless. The febrile symptoms, often very slight, pass off rapidly, and the second stage commences. The child regains to all appearances its usual health, except that there is no return of power to the muscles. In this state the child remains for some weeks, gradually passing into the third stage in which there may or may not be a partial recovery of power. It is seldom complete, if, indeed, ever. In the final stage the limit of recovery is reached; no hope for further improvement can be entertained, there only remains for the medical man to counteract the effect of the unbalanced muscles, and to apply such mechanical support as may relieve the patient in supplying power by means of springs, &c. One of the effects of the inflammatory action which causes the disease is to impair the nutrition of the limb. The circulation of the skin becomes poor, giving rise to much trouble in cold weather. It is very rash in the early stages of the disease to pronounce any definite opinion with regard to the amount of permanent damage that is likely to remain in after life. The more one sees of the disease the more one is struck with the impossibility of saying how much recovery can take place.

The pathology of the disease is very simple, consisting in inflammation and subsequent degeneration of the large nerve cells in the anterior columns of the cord. On the healthy condition of these cells in the cervical and lumbar enlargements depends the health of the muscles of the limbs, and destruction of these cells by inflammation at once reveals itself in loss of tone, and subsequently in the wasting of the muscles.

The manner in which groups of muscles are affected is often peculiar, having no regard to the peripheral nerve supply, so that we often see muscles having no relation to one another attacked. In the lower limb the muscles below the knee are most often affected, the peronei most, so that talipes-equino-varus is produced from the unbalanced action of the gastrocnemius. Sometimes the tibialis anticus is paralysed, and talipes valgus results, and talipes-calcaneus when the gastrocnemius is affected. In some cases all the muscles of the legs are affected, and total loss of power follows, the patient dragging himself along by his arms, trailing his useless legs behind him.

*The Treatment of the Disease.*—In the early stages rest and quiet are of paramount importance, with the exhibition of antipyretic drugs and low diet, and these must be continued for a considerable time. We can hardly hope to affect the inflammatory condition of the cord with the drugs at present known to us, and often the damage is done before the case comes under notice. In the later stages much may be done to preserve the nutrition of the muscles by massage and the application of the electric current, in the hope that some of the affected cells may recover their normal state. Careful observation by the interrupted and continued currents will, however, show that there is small hope of recovery in certain muscles, for after a time the reactions of degeneration set in, and permanent loss of irritability in the course of time. Children should be encouraged to make use of their limbs within a short time of the onset of the attack, and more especially to attempt to use those muscles which are affected. When a whole limb is affected it must be carefully kept warm and the skin stimulated by friction. To correct the deformities division of the tendons of the unbalanced muscles may be performed, and numerous mechanical devices, such as springs attached to the foot, &c., have been invented to assist and support the limb.

